# Artificial Intelligence: A Reliable Tool to Detect the Elongation of the Styloid Process

**DOI:** 10.7759/cureus.49541

**Published:** 2023-11-28

**Authors:** Jebarani Jeevitha S, Lokesh Kumar S, Pradeep Kumar Yadalam

**Affiliations:** 1 Department of Oral Medicine, Radiology, and Special Care Dentistry, Saveetha Dental College and Hospitals, Saveetha Institute of Medical and Technical Sciences (SIMATS) Saveetha University, Chennai, IND; 2 Department of Periodontology, Saveetha Dental College and Hospitals, Saveetha Institute of Medical and Technical Sciences (SIMATS) Saveetha University, Chennai, IND

**Keywords:** eagle's syndrome, technology, panoramic, radiography, algorithms, machine learning, artificial intelligence

## Abstract

Background

Eagle's syndrome is characterized by the anomalous elongation of the styloid process. This condition is usually identified through the manual evaluation of orthopantomogram (OPG) images, which is time-consuming and can have interobserver variability. The application of Artificial intelligence (AI) in radiology is gaining importance and interest in recent years. The application of AI in detecting styloid process elongation is less explored, advocating for research in the same arena.

Aim and objectives

The study aimed to evaluate the accuracy of artificial intelligence in detecting styloid process elongation in digital OPGs and to compare the performance of the three different AI algorithms with that of the manual radiographic evaluation by the radiologist.

Materials and methods

A total of 400 digital OPGs were screened, and linear measurements of the styloid process length (ImageJ software (National Institute of Health, Maryland, USA)) were done for the identification of styloid process elongation by a single calibrated observer to finally include a processed image dataset including 169 images of the elongated styloid process and 200 images of the normal styloid process. A machine learning approach was used to detect the styloid process elongation using the three different AI models: logistic regression, neural network, and Naïve Bayes algorithms in Orange software (University of Ljubljana, Slovenia). Performance evaluation was done using the accuracy, sensitivity, specificity, precision, recall, F1 score, and AUC-ROC (area under the receiver operating characteristic) curve.

Results

Logistic regression and neural network algorithms depicted the highest accuracy of 100% with no false positives or false negatives, securing a score of 1.000 for all the metrics. However, the Naïve Bayes model demonstrated a fairly considerable accuracy, classifying 49 false positive images and 59 false negative images with an AUC (area under the curve) score of 78 %. Nevertheless, it performed better than random guessing.

Conclusion

Logistic regression and neural network algorithms accurately detected styloid process elongation similar to that of manual radiographic evaluation. The Naïve Bayes algorithm did not perform an accurate classification yet performed better than random guessing. AI holds a promising scope for its application in automatically detecting styloid process elongation in digital OPGs.

## Introduction

The styloid process is a slender, bony projection situated anteriorly to the stylomastoid foramen, typically measuring between 20 to 25 millimeters in length [1]. However, when this structure elongates beyond the usual range, extending past 30 millimetres [2], it can give rise to a spectrum of clinical symptoms. This medical condition is well-recognized as Eagle's syndrome.

Eagle's syndrome is a relatively rare but potentially debilitating condition [3] characterized by the abnormal elongation of the styloid process. When this elongation occurs, it can lead to a variety of distressing symptoms [4]. Patients afflicted by Eagle's syndrome may experience persistent neck and cervicofacial pain and dysphagia, among other discomforts [5,6]. These symptoms can be chronic and significantly impact a person's quality of life, often necessitating medical attention and intervention.

The diagnosis of Eagle's syndrome typically involves imaging studies, with digital orthopantomograms (commonly known as panoramic radiographs) being a valuable tool for evaluating the styloid process [6]. Traditional diagnostic methods, reliant on manual interpretation of radiographic images, are prone to variability among healthcare professionals and can be time-consuming. However, artificial intelligence (AI)-driven approaches have emerged as a promising solution to address these limitations.

In recent years, there has been a notable advancement in the field of medical imaging, specifically in the application of AI and machine learning (ML) techniques [7]. These technologies have been increasingly integrated into medical practice to aid in interpreting and analyzing complex medical images. ML algorithms can be trained to recognize patterns, anomalies, and specific structures within medical images, potentially enhancing diagnostic accuracy and efficiency.

The integration of AI and ML technologies into medical imaging has marked a significant advancement in the field of healthcare [7]. These technologies can revolutionize how clinicians approach diagnosing and managing various medical conditions, including rare but impactful disorders. More experienced radiologists tend to have more diagnostic accuracy compared to the less experienced ones. However, even a well-qualified radiologist may miss an important radiographic finding or misinterpret the radiograph due to mental or eye fatigue, which may affect the final diagnostic decision [8]. The incorporation of AI in radiographic interpretation may reduce the workload for the radiologist as well as reduce the time taken for radiographic interpretation.

The utilization of AI in this context holds great promise for improving the diagnostic process related to Eagle's syndrome. By automating the detection of styloid process elongation in digital orthopantomograms, this technology can enhance the efficiency of clinical assessments, reduce variability in diagnoses, and ultimately improve patient care. Additionally, it opens up new avenues for the application of AI in the field of radiology. It may serve as a model for integrating ML in diagnosing other anatomical abnormalities and medical conditions.

Hence, this study attempts to shed light on the feasibility and efficacy of AI-driven diagnostic tools, paving the way for enhanced diagnostic capabilities in radiology and beyond. Ultimately, the findings of this study may contribute to improved patient care and the continued integration of AI into modern healthcare practices.

This study aimed to evaluate the accuracy and effectiveness of AI algorithms in detecting styloid process elongation in digital orthopantomograms. The research objectives were to assess the accuracy of AI algorithms in identifying cases of styloid process elongation in OPGs and to compare the accuracy of different AI algorithms with the manual evaluations by the radiologist. This involved training ML models to recognize and differentiate between normal styloid processes and elongated ones based on patterns and features extracted from these radiographic images.

## Materials and methods

This present study is a digital orthopantomographic study done utilizing the digital panoramic radiographs retrieved from the radiological archives of Saveetha Dental College and Hospitals, Chennai, India. The present study has been waived for ethical clearance by the Institutional Ethical Committee with Reference number IHEC/SDC/WAIVER CERT-2201/23/02. The orthopantomograms depicting the styloid process on both the right and left sides of patients above 18 were included. However, those with size and shape distortions and radiographs of patients under 18 years of age were excluded.

A total of 400 orthopantomograms (convenient sampling) imaged using the same digital panoramic machine, Carestream CS8100SC (Carestream Dental LLC, Atlanta, Georgia, United States), were selected for evaluating the styloid process by a single calibrated observer based on the abovementioned inclusion and exclusion criteria. The length of the styloid process was measured utilizing the two-point linear measurements done on ImageJ software (National Institute of Health, Maryland, USA) [9] from the point where it left the temporal bone to its tip according to the method described by Ilgüy et al*.* [10] on the digital orthopantomograms to determine the presence or absence of styloid process. Only 20 radiographs were evaluated in a single day to eliminate possible bias or errors due to examiner fatigue. Based on the preliminary radiographic measurements, the orthopantomographic scans were segregated to have a normal styloid process, unilateral (right or left) elongation, and bilateral elongation (both left and right). The study group included the images with an elongated styloid process either unilaterally or bilaterally and the control group included the images with a normal styloid process. Such segregated images were further processed by image preparation, image embedding, and a ML approach. For size standardization, the images were prepared using a cropping tool (snipping tool in Microsoft Windows 11 OS software (Microsoft Corporation, Redmond, Washington, United States)) [11]. A single image with bilateral elongation of the styloid process was considered two images after cropping. The dataset was divided into 50 percent training and 50 percent testing subsets. The results from the ML approach were compared with that of the manual radiographic evaluation.

ML approach

**Figure 1:**
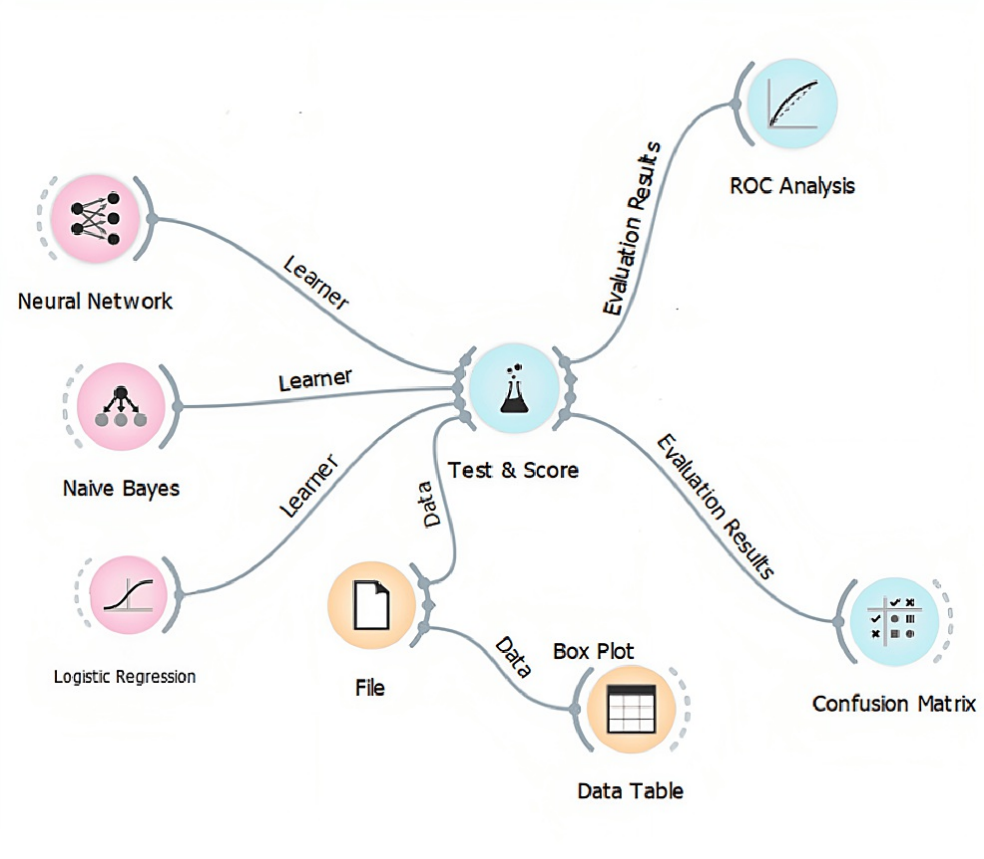
Widgets in Orange workflow The workflow figure was generated by the authors in Orange Software, Version 3.35.0, (Released 2023, University of Ljubljana, Slovenia). ROC analysis: Receiver operating characteristic analysis

The research employed an ML approach to analyze the annotated images. The workflow involved the use of the Orange Software, Version 3.35.0, (Released 2023, University of Ljubljana, Slovenia) (cross-platform computer software) [12], an open-source data visualization AI tool, which supports various data processing and ML tasks, including data loading, transformation, visualization with user interaction, model inference, and model visualization [13]. Orange is a component-based visual programming software package for data visualization, ML, data mining, and data analysis. The workflow of the ML approach is depicted in Figure 1.

Orange is a data mining framework that provides clustering, classification, and interactive data and model visualization components. It also has an image analytics add-on that includes image-specific extensions. The dataset with cross-validation was performed on three ML models (algorithms), namely logistic regression, neural networks, and Naïve Bayes, to obtain robust results.

ML algorithms used

*Logistic Regression* 

This classification algorithm is suitable for categorical response variables and aims to establish the relationship between features and the probability of a particular binary outcome, such as whether an image is a normal styloid or elongated styloid process [14].

Neural Network 

This step builds a neural network model to categorize the images. Neural networks are ML models that can learn complex relationships between predictors and outcomes [14].

Naïve Bayes 

This step builds a Naïve Bayes model to classify the images. Naïve Bayes is a probabilistic classifier that assumes that the predictors are independent [14].

Confusion matrix

A confusion matrix is a table that can be used to summarize the performance of a classifier. It shows the number of true positives (TP), false positives (FP), true negatives (TN), and false negatives (FN). It can be used to calculate precision, recall, and F1 score. It can also be used to visualize the performance of a classifier.

The confusion matrix table is divided into four quadrants, namely 1) TP: the number of images that were elongated styloid process and were correctly predicted as elongated styloid process by the algorithm, 2) FP: the number of images that were actually normal styloid process but were incorrectly predicted as elongated styloid process by the algorithm, 3) TN: The number of images that were actually normal styloid process and were correctly predicted as normal styloid process by the algorithm, 4) FN: The number of elongated styloid process images that were incorrectly predicted as normal styloid process by the algorithm.

Accuracy

Accuracy is the ability of an algorithm to differentiate between diseased and normal cases. The accuracy of an ML algorithm is calculated using the formula (TP + TN) / (TP + TN + FP + FN).

Precision

Precision is the fraction of TP out of all predicted positives (TP + FP). The formula for calculating precision is TP / (TP + FP).

Recall

Recall is the fraction of TP out of all actual positives (TP + FN). The formula for calculating recall is TP / (TP + FN).

A good classifier should have high precision and recall. Ideally, a good classifier would have both precision and recall of 1, meaning there are no false positives (FP) or false negatives (FN). However, this is not always possible, so we need a metric that takes both precision and recall into account

F1 score

The F1 score is a metric that considers both precision and recall. It is defined as 2 * (precision * recall) / (precision + recall). The F1 score is always between 0 and 1, with 1 being the best possible score. A score of 0.5 means the classifier is not doing better than random guessing.

Performance evaluation

The primary performance metric for evaluating the classification models was the AUC-ROC curve (area under the receiver operating characteristic curve), a statistical metric used to evaluate the performance of a binary classification model [14]. It quantifies the model's ability to distinguish between two classes, typically the positive class (cases of interest) and the negative class (non-cases).

The ROC (receiver operating characteristic) curve [15] is a graphical representation of a classification model's performance across different discrimination thresholds. It plots two essential metrics, namely true positive rate (TPR) or sensitivity and false positive rate (FPR)

TPR or Sensitivity

This measures the proportion of positive cases the model correctly identifies as positive. It's the ratio of true positives to the total number of actual positives. TPR is calculated using the formula TP / (TP + FN).

FPR

This measures the proportion of actual negative cases the model incorrectly identifies as positive. It's the ratio of false positives to the total number of actual negatives.

Specificity

Specificity is the ability of the ML model to identify the true negative cases. It is otherwise called a true negative rate. Specificity is calculated using the formula TN / (TN + FP).

AUC (area under the curve) calculation

The AUC-ROC metric quantifies the area under the ROC curve and offers insights into the model's discrimination ability. It ranges from 0 to 1, and the general interpretation is given in the following manner. AUC = 0.5: the model is no better than random guessing. AUC > 0.5: the model has some ability to discriminate between the classes, with a higher AUC indicating better discrimination. AUC = 1: the model has perfect discrimination; it correctly identifies all positive cases while never misclassifying a negative case.

It summarizes a model's ability to make accurate classifications across different threshold levels. For instance, a high AUC-ROC score in medical diagnostics suggests that the model correctly identifies individuals with elongated styloid processes (sensitivity) while minimizing false positives (specificity), which is crucial for avoiding unnecessary treatments or interventions.

## Results

The study included a total of 400 orthopantomogram images for the evaluation of the styloid process. It was found that 99 images had an elongated styloid process, and 301 images had the normal styloid process. Among the 301 images with the normal styloid process, only 100 images were selected to be included for further processing. The gender distributions of the patients' orthopantomogram images that were finally selected are depicted in Table 1.

**Table 1:** Gender distribution of patients

Styloid process	Males (n)	Females (n)	Total
Normal styloid process	50	50	100
Elongated styloid process	48	51	99
Total	98	101	199

The dataset was further delineated among the images with elongated styloid processes (99 images) and was further segregated between bilateral elongated styloid processes, left side alone, and right side alone. As mentioned earlier, during image preparation, the single image with bilateral elongation of the styloid process was considered two images after cropping. Hence, the final cropped images with the elongated styloid process accounted for 169 images (E1) (70 x 2 = 140 (bilateral elongation) + 13 (left side elongation) + 16 (right side elongation) = 169). Similarly, the final included images with normal styloid process accounted for 200 images after cropping (N1) (100 x 2 = 200).

The logistic regression model predicted all the images accurately, indicating no false positives or negatives, and was consistent with the manual radiographic evaluation. The accuracy of the logistic regression model was 100%. The confusion matrix of the logistic regression model is depicted in Table 2.

**Table 2:** Confusion matrix for logistic regression showing the proportions of predicted elongated styloid and predicted normal styloid cases compared to actual cases

Predicted				
Actual	Logistic regression	Elongated styloid (E1)	Normal styloid (N1)	Σ
	Elongated styloid (E1)	100.0%	0.0%	169
	Normal styloid (N1)	0.0%	100.0%	200
	Σ	169	200	369

The neural network algorithm showcased similar performance, correctly classifying the 169 images as having elongated styloid process and 200 images as having normal styloid process. This model showed 100% accuracy with no false positives or negatives, indicating an accurate prediction for all the test data images similar to the manual radiographic evaluation. Table 3 shows the confusion matrix of the neural network algorithm.

**Table 3:** Confusion matrix for neural network showing the proportions of predicted elongated styloid and predicted normal styloid cases compared to actual cases

Predicted				
Actual	Neural network	Elongated styloid (E1)	Normal styloid (N1)	Σ
	Elongated styloid (E1)	100.0%	0.0%	169
	Normal styloid (N1)	0.0%	100.0%	200
	Σ	169	200	369

The Naïve Bayes algorithm classified 185 images as having an elongated styloid process and 184 images as having a normal styloid process. It correctly classified 113 images as having an elongated styloid process (TP) and 151 as having a normal styloid process (TN). However, it misclassified 49 images as having an elongated styloid process (FP) and 56 images as having a normal styloid process (56), which were not in agreement with the manual radiographic evaluation. The confusion matrix of the model is depicted in Table 4.

**Table 4:** Confusion matrix of the Naïve Bayes with the proportion of predicted versus actual elongated styloid process and normal styloid process

Predicted				
Actual	Naïve Bayes	Elongated styloid (E1)	Normal styloid (N1)	Σ
	Elongated styloid (E1)	67.0%	33.0%	169
	Normal styloid (N1)	24.5%	75.5%	200
	Σ	185	184	369

The accuracy of the Naïve Bayes algorithm was calculated to be 0.715. This means the Naïve Bayes algorithm correctly classified 71.5% of the images. The precision and recall of this model were 0.697 and 0.668. Table 5 shows the test and score results for all three algorithms tested in this study.

**Table 5:** Test and score model performance of all three algorithms AUC: Area under the curve; F1: F1 score

Model	AUC	Accuracy	F1	Precision	Recall
Neural network	1.000	1.000	1.000	1.000	1.000
Naïve Bayes	0.782	0.715	0.682	0.697	0.668
Logistic Regression	1.000	1.000	1.000	1.000	1.000

**Figure 2:**
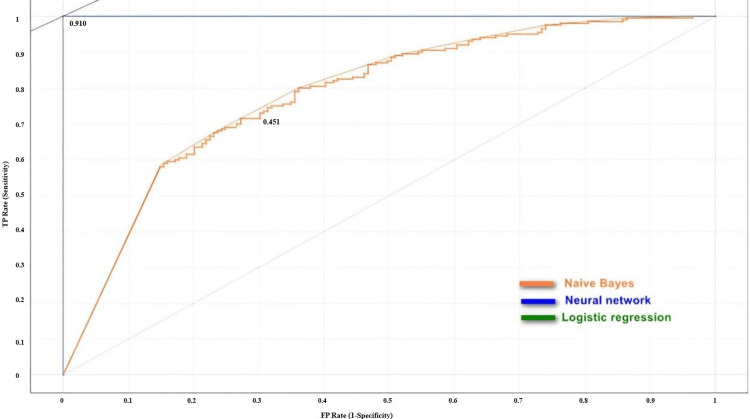
ROC analysis of the performance of all three algorithms for detecting the normal styloid process ROC analysis: Receiver operating characteristic analysis; TP Rate: True positive rate; FP Rate: False positive rate

Figure 2 shows the ROC analysis that revealed the performance of the three algorithms for detecting normal styloid process from the test data. The curve starts at the point (0,0), where the algorithm did not correctly predict images. As the curve moves upwards and to the right, it indicates that the algorithm is becoming more accurate. When the curve reaches its maximum point at (1,1), the algorithm correctly predicts all images. The diagonal dotted line in the graph represents the performance of a random classifier. The full diagonal line or the iso-performance line represents the threshold for achieving consistent performance. If we shift the threshold higher or further to the left, it becomes unattainable for the learner.

Conversely, moving it lower or to the right reduces the overall performance. The ROC analysis showed that the logistic regression and the neural network algorithms had superior accuracy in correctly identifying the normal styloid process and were never misclassified. However, the Naïve Bayes algorithm model was fairly accurate as it positioned itself approximately midway between the performance line of a random classifier and the iso-performance line.

**Figure 3:**
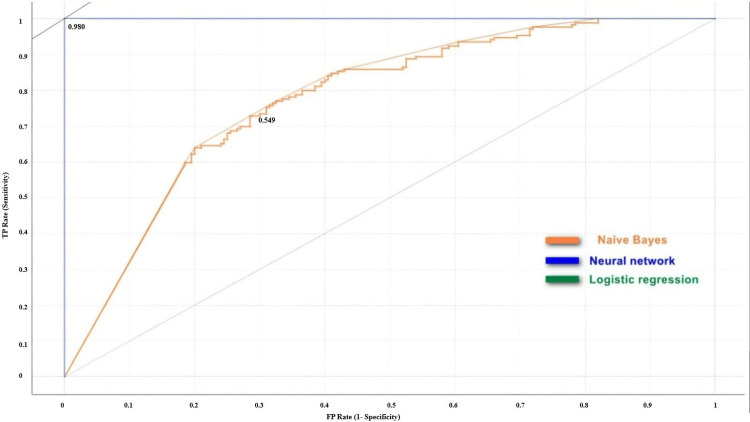
ROC analysis of the performance of all three algorithms in detecting the elongated styloid process ROC analysis: Receiver operating characteristic analysis; TP Rate: True positive rate; FP Rate: False positive rate

Similarly, Figure 3 shows the performance of the algorithms for detecting the elongated syloid process from the test data. The ROC analysis displayed that the logistic regression and neural network models had similar superior accuracy in identifying the elongated styloid process and never misclassified. However, the Naïve Bayes algorithm model demonstrated a reasonable level of accuracy by having its position in the middle between the performance line of a random classifier and the iso-performance line.

## Discussion

The present study attempted to evaluate the efficiency of AI as a tool in detecting the elongation of styloid processes in digital orthopantomogram images. To the best of our knowledge, this is the first study to explore AI's ability to be a reliable tool in detecting styloid process elongation. The dataset was divided into 50 percent training and 50 percent testing subsets, with cross-validation performed on three different ML models to obtain robust results. Logistic regression, neural networks, and Naïve Bayes algorithms were utilized for classification tasks, each offering unique advantages.

The current study revealed that the prevalence of styloid process elongation was 24.75% (99 out of 400), which is in accordance with the studies conducted by Deeksheetha et al. [16], whose study showed the elongation of the styloid process on the right and left side to be 35%. Males and females had a similar prevalence of elongated styloid process in the present study which was in agreement with the study by Divyadharshini and Vadivel [17].

Recently, AI has gained attention and importance in its incorporation into healthcare, especially radiology. AI-based techniques like texture analysis, including Grey Level Run Length 41 Matrix (GLRLM), Grey Level Co-occurrence Matrix (GLCM), wavelet analysis, etc., are utilized for the identification of pathologies in radiographic images [18]. Similarly, other measurement-based algorithms are also utilized.

Logistic regression and neural network algorithms demonstrated 100 % accuracy with no false positives or false negatives in their classification of the images, which was in agreement with that of manual radiographic evaluation. They also secured an AUC score of 1, indicating the ability of perfect discrimination to identify the elongated styloid process while never misclassifying a normal styloid process. This high level of accuracy signifies that both models are very effective in detecting styloid process elongation. This was in accordance with the previous study by Pourhomayoun and Shakibi [19], where they designed and developed a predictive model based on AI and ML algorithms to determine the health risk and predict the mortality risk of patients with coronavirus disease 2019 (COVID-19). They used a dataset of more than 2,670,000 laboratory-confirmed COVID-19 patients from 146 countries worldwide, including 307,382 labeled samples. Their results demonstrated 89.98% overall accuracy in predicting the mortality rate. The current study showed a precision, recall, and F1 score of 1, indicating an accurate and very good performance detecting styloid process elongation. The higher the precision value, the better the models' ability to correctly predict positive instances, and the higher the recall value, the better the models' ability to correctly predict negative instances. The F1 score indicates the measure of the balance between the above-mentioned two entities [12]. The two algorithm models had a similar superior balance between precision and recall. 

Nevertheless, the choice of which table to use depends on the application's specific requirements. If differentiating the elongated styloid process from the normal one is required, logistic regression alone is sufficient. But for more computational tasks, the neural network model is the model of choice to get results with high accuracy because the decision boundary is nonlinear in the neural network algorithm compared to the logistic regression model, which is linear, making the model more flexible and thus more susceptible to over-fitting compared to logistic regression. However, the neural network algorithm is more complex and computationally expensive than the logistic regression table. A previous study by Tu [20] provided a new alternative to logistic regression, the most commonly used method for developing predictive models for dichotomous healthcare outcomes. They stated that neural networks offer several advantages, including requiring less formal statistical training, the ability to implicitly detect complex nonlinear relationships between dependent and independent variables, the ability to detect all possible interactions between predictor variables, and the availability of multiple training algorithms.

However, The Naïve Bayes algorithm did not perform an accurate classification during the testing, with an accuracy of 71.5%, and differed from the results of the manual radiographic evaluation for the rest 28.5% of the images. The confusion matrix table revealed a higher percentage of false positives and false negatives. This is because the Naïve Bayes algorithm assumes that all of the features are independent of each other. This assumption may not be true in the case of images, where the features may be correlated with each other. This was in accordance with the previous study by Saritas and Yasar [21], who used AI to estimate the potential of breast cancer by taking advantage of anthropometric data and collecting routine blood analysis parameters. Their performance evaluation was based on correct and incorrect data classification examples using the artificial neural network (ANN) and Naïve Bayes classification algorithm. Their results were classified with an accuracy of 86.95 % with ANN and 83.54 % with Naïve Bayes algorithm.

Overall, the results of the current study demonstrated that the Naïve Bayes algorithm is not very accurate for classifying the elongated styloid process from the normal styloid process. However, it is still better than random guessing, which would have resulted in a confusion matrix with 50% true positives and 50% false positives.

The test results showed that the logistic regression and neural network models had the highest accuracy and were consistent with the manual radiographic evaluation, with an AUC of 100%. Naïve Bayes was fairly accurate, with an AUC of 78%. This difference in accuracy may be due to the complexity of the task, the nature of the data, and the capabilities of the algorithms to handle it. Logistic regression and neural networks can learn complex relationships and patterns, while Naïve Bayes may struggle with more complex dependencies among features.

The results were evaluated by performance criteria such as accuracy, precision, recall, F1 score, sensitivity, specificity, and AUC-ROC. These metrics were used to identify areas where the model can be improved. For example, the F1 score for Naïve Bayes algorithm was lower than the F1 scores for logistic regression and neural networks, suggesting that Naïve Bayes model may not be as good at capturing the trade-off between precision and recall.

Hence, the present study demonstrated a good performance from two algorithm models out of the three evaluated AI algorithms and was consistent with the manual evaluations. However, the present study involved a limited dataset, including 369 orthopantomogram images of patients visiting a single center. Future studies with a larger dataset and the inclusion of the detection of different calcification patterns of the styloid process are warranted to evaluate AI's performance in such complex tasks.

## Conclusions

The findings of the present study demonstrated that logistic regression and neural network algorithms have the highest accuracy in detecting elongated styloid processes in digital orthopantomogram images, which was consistent with the manual radiographic evaluation. Also, the Naïve Bayes algorithm model showed a fairly considerable performance in the classification. In conclusion, AI holds a highly promising value and scope in detecting elongated styloid processes in digital orthopantomograms, enabling automatic detection in the future.
